# The Role of cGMP on Adenosine A_*1*_ Receptor-mediated Inhibition of Synaptic Transmission at the Hippocampus

**DOI:** 10.3389/fphar.2016.00103

**Published:** 2016-04-22

**Authors:** Isa Pinto, André Serpa, Ana M. Sebastião, José F. Cascalheira

**Affiliations:** ^1^CICS-UBI – Health Sciences Research Center, University of Beira InteriorCovilhã, Portugal; ^2^Institute of Pharmacology and Neurosciences, Faculty of Medicine, University of LisbonLisboa, Portugal; ^3^Institute of Molecular Medicine, University of LisbonLisboa, Portugal; ^4^Department of Chemistry, University of Beira InteriorCovilhã, Portugal

**Keywords:** adenosine A_1_ receptor, cGMP, hippocampus, synaptic transmission, soluble guanylyl cyclase, protein kinase G

## Abstract

Both adenosine A_1_ receptor and cGMP inhibit synaptic transmission at the hippocampus and recently it was found that A_1_ receptor increased cGMP levels in hippocampus, but the role of cGMP on A_1_ receptor-mediated inhibition of synaptic transmission remains to be established. In the present work we investigated if blocking the NOS/sGC/cGMP/PKG pathway using nitric oxide synthase (NOS), protein kinase G (PKG), and soluble guanylyl cyclase (sGC) inhibitors modify the A_1_ receptor effect on synaptic transmission. Neurotransmission was evaluated by measuring the slope of field excitatory postsynaptic potentials (fEPSPs) evoked by electrical stimulation at hippocampal slices. N6-cyclopentyladenosine (CPA, 15 nM), a selective A_1_ receptor agonist, reversibly decreased the fEPSPs by 54 ± 5%. Incubation of the slices with an inhibitor of NOS (L-NAME, 200 μM) decreased the CPA effect on fEPSPs by 57 ± 9% in female rats. In males, ODQ (10 μM), an sGC inhibitor, decreased the CPA inhibitory effect on fEPSPs by 23 ± 6%, but only when adenosine deaminase (ADA,1 U/ml) was present; similar results were found in females, where ODQ decreased CPA-induced inhibition of fEPSP slope by 23 ± 7%. In male rats, the presence of the PKG inhibitor (KT5823, 1 nM) decreased the CPA effect by 45.0 ± 9%; similar results were obtained in females, where KT5823 caused a 32 ± 9% decrease on the CPA effect. In conclusion, the results suggest that the inhibitory action of adenosine A_1_ receptors on synaptic transmission at hippocampus is, in part, mediated by the NOS/sGC/cGMP/PKG pathway.

## Introduction

Adenosine A_1_ receptor activation and cyclic guanosine monophosphate (cGMP) have similar actions at the nervous system. Both decrease neurotransmitter release and synaptic transmission, including excitatory synaptic transmission at the hippocampus (Dunwiddie and Hoffer, 1980; Nordström and Bartfai, 1981; Prast and Philippu, 2001; Feil and Kleppisch, 2008; Serpa et al., 2009; Dias et al., 2013), protect against neurotoxic insults (Montoliu et al., 2001; Ribeiro et al., 2003; Orio et al., 2007; Serpa et al., 2015b) and modulate synaptic plasticity, including long-term depression at the hippocampus (Santschi et al., 2006; Feil and Kleppisch, 2008; Dias et al., 2013). However, the relationship between adenosine A_1_ receptors and cGMP remains to be clarified.

Cyclic guanosine monophosphate is produced by the action of guanylyl cyclases (GC), which are a family of enzymes that catalyze the conversion of GTP to cGMP. There are two types of GC, the soluble form (sGC), activated by nitric oxide (NO), and the particulated form (pGC), which is a receptor for extracellular ligands such as natriuretic peptides (Lucas et al., 2000). cGMP is degraded by phosphodiesterases (PDEs), which catalyze its hydrolysis (Kleppisch, 2009). The main effector of cGMP is protein kinase G (PKG), which associates with the scaffold protein GKAP. GKAP targets different PKG isoforms to different subcellular compartments (Schlossmann and Desch, 2009) where the PKG isoforms phosphorylate specific substrate proteins.

The adenosine A_1_ receptor is the most abundant adenosine receptor in the central nervous system (Ribeiro et al., 2003). It is coupled to G_i/o_ proteins, inhibiting adenylyl cyclase and consequently decreasing cyclic adenosine monophosphate (cAMP) formation and protein kinase A (PKA) activation (Fredholm et al., 2001; Serpa et al., 2015a). Furthermore adenosine A_1_ receptor also regulates phospholipase C activity (Cascalheira and Sebastião, 1998; Cascalheira et al., 2002), inhibits N- and P/Q-type calcium channels (Ambrósio et al., 1997) and activates inwardly rectifying potassium channels (Takigawa and Alzheimer, 2002).

Previous studies have shown that NO donors inhibit synaptic transmission in slices of hippocampus, and this inhibition was blocked by adenosine A_1_ receptor antagonists (Boulton et al., 1994; Broome et al., 1994). Later it was found that the inhibitory effect of NO on synaptic transmission involves adenosine release to the extracellular medium and is unaffected by inhibition of sGC (Arrigoni and Rosenberg, 2006). On the other hand, NO was shown to enhance the inhibitory effect of 2-chloroadenosine (CADO) in synaptic transmission and this effect of NO was blocked by inhibitors of sGC (Fragata et al., 2006). Our group recently observed that activation of the adenosine A_1_ receptor increased cGMP levels in the hippocampus (Serpa et al., 2014). However, whether the inhibitory effect of adenosine A_1_ receptor on synaptic neurotransmission is mediated by cGMP is yet unknown. To answer this question we tested if blockade of components of the cGMP signaling pathway would modify the inhibitory effect of adenosine A_1_ receptors in synaptic transmission at rat the hippocampus.

## Materials and Methods

### Hippocampal Slice Preparation

Young adult male and female Wistar rats (8–10 weeks old) were handled according to European Community guidelines and Portuguese law concerning animal care, and anesthetized with Isoflurane before decapitation. Experiments were approved by the ethical committee of the Institute of Molecular Medicine and Faculty of Medicine, University of Lisbon. The brain was quickly removed and the hippocampi dissected under ice-cold Krebs–Henseleit solution with the following composition (mM): NaCl 118, NaHCO_3_ 25, KCl 4.7, glucose 11.6, KH_2_PO_4_ 1.2, MgSO_4_ 1.2, CaCl_2_ 1.3, gassed with O_2_ (95%), and CO_2_ (5%) (pH = 7.4). After hippocampal dissection, transverse slices (400 μm thick) were cut with a McIlwain tissue chopper and allowed to recover in gassed Krebs–Henseleit solution at room temperature (22–25°C) for at least one hour (1h) before use. For recordings, the slices were transferred to a recording chamber (1 ml capacity) and continuously perfused at 3 ml/min with Krebs–Henseleit buffer maintained at 32°C with a TC-202A temperature controller. Drugs to be tested were applied to the perfusion solution. Whenever testing the effect of a drug (test drug) in the presence of another drug (modifier drug), the test drug was only added to the bath after allowing a full effect of the modifier drug, i.e., the fEPSP slope had to return to a stable value for at least 10min before the second addition of the test drug to the slices. The usual procedure was to apply the modifier drug 1 h after starting the washout of the first application of test drug and for at least 30 min before application of the test drug in its presence.

### Recording Field Excitatory Post-synaptic Potentials (fEPSP)

Field excitatory postsynaptic potentials (fEPSPs) were recorded through an extracellular microelectrode (2–8 MΩ resistance, filled with a 4 M NaCl solution) placed in stratum radiatum of Cornus Ammonis 1 (CA1). Stimulation (rectangular 0.1 milliseconds pulses), was delivered once every 15 s through a bipolar concentric wire electrode positioned in the Schaffer collaterals-commissural fibers, in the stratum radiatum near the CA3–CA1 border. The intensity of stimulus (80–200 μA intensity) was adjusted to obtain a large fEPSP with a minimum population spike contamination. To avoid supramaximal stimulation, the stimulus intensity was also adjusted to obtain a fEPSP slope within 50–80% of its maximum value under supramaximally stimulating conditions. When using inhibitors (modifier drug) of the NOS/sGC/PKG pathway, whenever the effect of the inhibitor alone produced an increase of the fEPSP slope higher than 80% of its maximum value, after the effect of the antagonist stabilized and prior to the second application of CPA (test drug), the intensity of the stimulus was also adjusted to obtain a baseline roughly identical to that obtained before the first application of CPA. Extracellular recordings were obtained with an Axoclamp 2B amplifier and digitized (using a National Instruments BNC 2120 interface at a sample interval of 50 μs (20 kHz)). Individual fEPSPs were monitored, and averages of eight consecutive responses were recorded and analyzed through the LTP 230d software (Anderson and Collingridge, 2001). Responses were quantified as the slope of the initial phase of the averaged fEPSPs, since slope measures are considered a more accurate measure of fEPSP magnitude than the amplitude, due to eventual contamination by the population spike.

### Drugs

N6-cyclopentyladenosine (CPA) was purchased from Tocris, (9*S*,10*R*,12*R*)-2,3,9,10,11,12-Hexahydro-10-methoxy-2,9-dimet-hyl-1-oxo-9,12-epoxy-1*H*-diindolo[1,2,3-*fg*:3′,2′,1′-*kl*]pyrrolo[3,4-*i*][1,6]benzodiazocine-10-carboxylic acid methylester (KT5823) was from Santa Cruz Biotecnology, 1H-[1,2,4]oxadiazole[4,3-a]quinoxalin-1-one (ODQ) and NG-nitro-L-arginine methylester (L-NAME) were from Sigma and Adenosine deaminase (ADA) was from Roche. All other reagents used were from analytical grade.

### Data Analysis and Statistics

Data was analyzed through the GraphPad Prism 6.0 software and expressed as mean ± SEM from n independent experiments. To allow comparisons between different experiments slope values were normalized, taking as 100% the averaged five values obtained immediately before applying the test drug(s). The significance of the differences between the means obtained in two different conditions, or when comparing means with zero, was evaluated by Student’s *t*-test, where the paired Student’s *t*-test was used whenever evaluating the significance of differences between two conditions tested in a paired way in the same experiment. Statistically significant differences were considered significant for values of *P* < 0.05. To compare the effect of CPA, in the absence and in the presence of a test drug, across gender, two-way ANOVA was used, followed by least significant difference (LSD) *post hoc* test and values of *P* < 0.05 were considered to represent statistically significant differences. Statistical power (Pw) of significance tests used, was calculated retrospectively using the PASS 14 Power Analysis and Sample Size Software (NCSS, LLC. Kaysville, UT, USA).

## Results

### Adenosine A_1_ Receptor Activity Is Dampened by a NOS Antagonist

**FIGURE 1:**
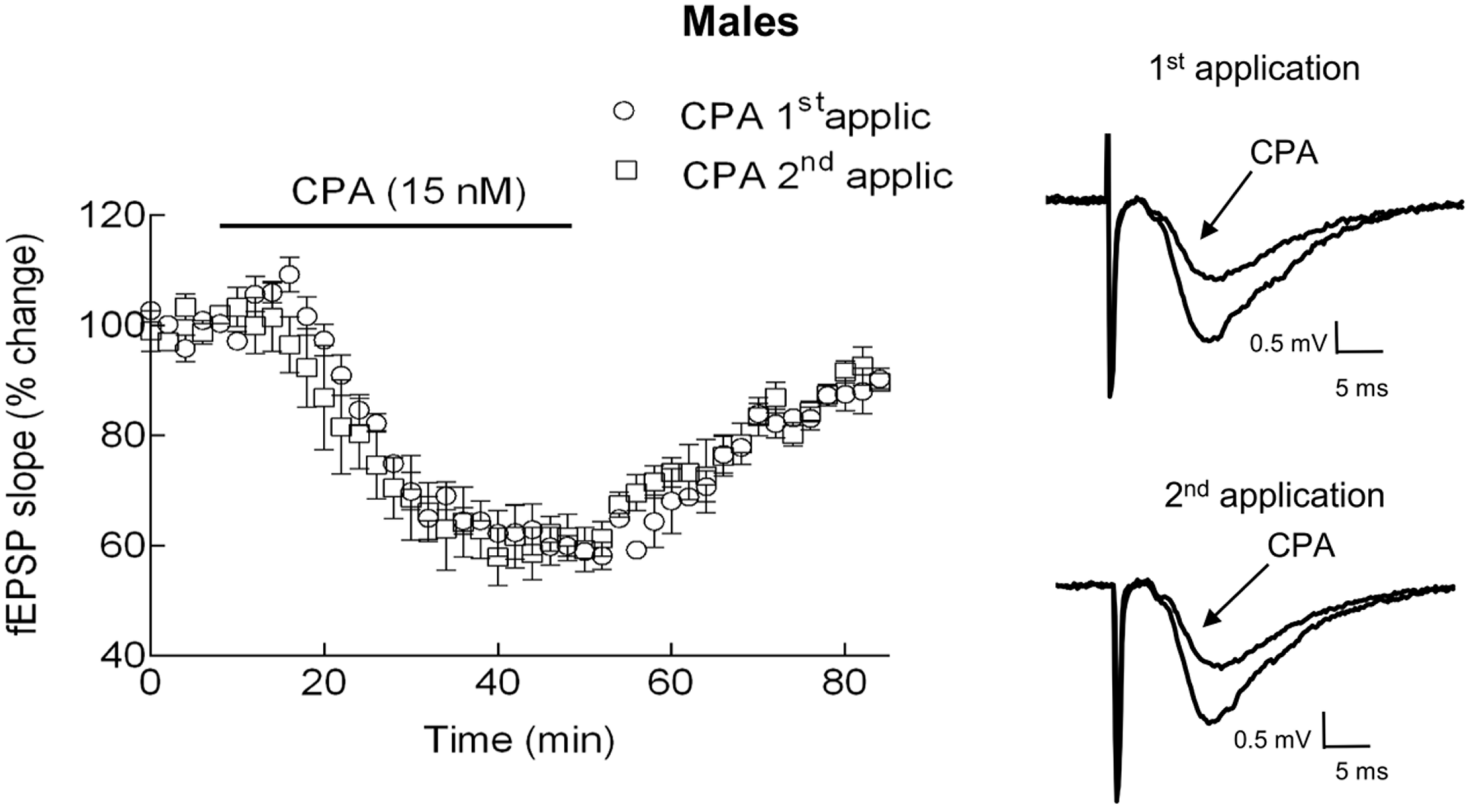
**Effect of CPA (15 nM) on the slope of fEPSPs in hippocampal slices of male rats. (Left)**: Superimposition of averaged time course changes of fEPSP slope induced by two consecutive applications of CPA (15 nM) separated by 90 min, in the absence of any additional drugs. Each point in the ordinates corresponds to the average ± SEM of fEPSP slopes, normalized for its value before addition of CPA, of three independent experiments. The horizontal bar indicates the time of perfusion with CPA. Right panel: Representative traces of average of fEPSPs recorded before and after 40 min application of CPA (15 nM), corresponding to the first **(upper)** or to the second **(lower)** application of CPA, are shown. The effect of CPA was obtained after 40 min of exposure to the drug, when the CPA effect stabilized. No significant differences were observed between the two applications of CPA (*P* > 0.05, paired Student’s *t*-test).

To allow comparisons of the effects of an agonist of adenosine A_1_ receptor in the absence and presence of a modifier drug in the same slice, we first tested if two consecutive applications of the adenosine A_1_ receptor selective agonist, 8-cyclopentyladenosine (CPA), separated by 90 min, caused a similar inhibition of evoked fEPSPs. CPA was used at a concentration (15 nM) previously shown to be selective for adenosine A_1_ receptor at hippocampal slices (Sebastião et al., 1990). As illustrated in **Figure 1**, no significant differences (*P* > 0.05, paired Student’s *t*-test; *P*w = 6%), between the fEPSP inhibition caused by the two consecutive applications of CPA were detected. Indeed, the first application of CPA (15 nM) decreased the fEPSP slope by 40 ± 2% (*n* = 3) whereas the second application decreased it by 41 ± 2% (*n* = 3); the time course of the inhibition as well as the washing out of the drug effect was also similar for each of the applications (**Figure 1**). Therefore, in the subsequent experiments, the effect of the 1^st^ application of CPA (15 nM) was used as internal control, when testing the effect of a second application of CPA in the presence of any other drug.

**FIGURE 2:**
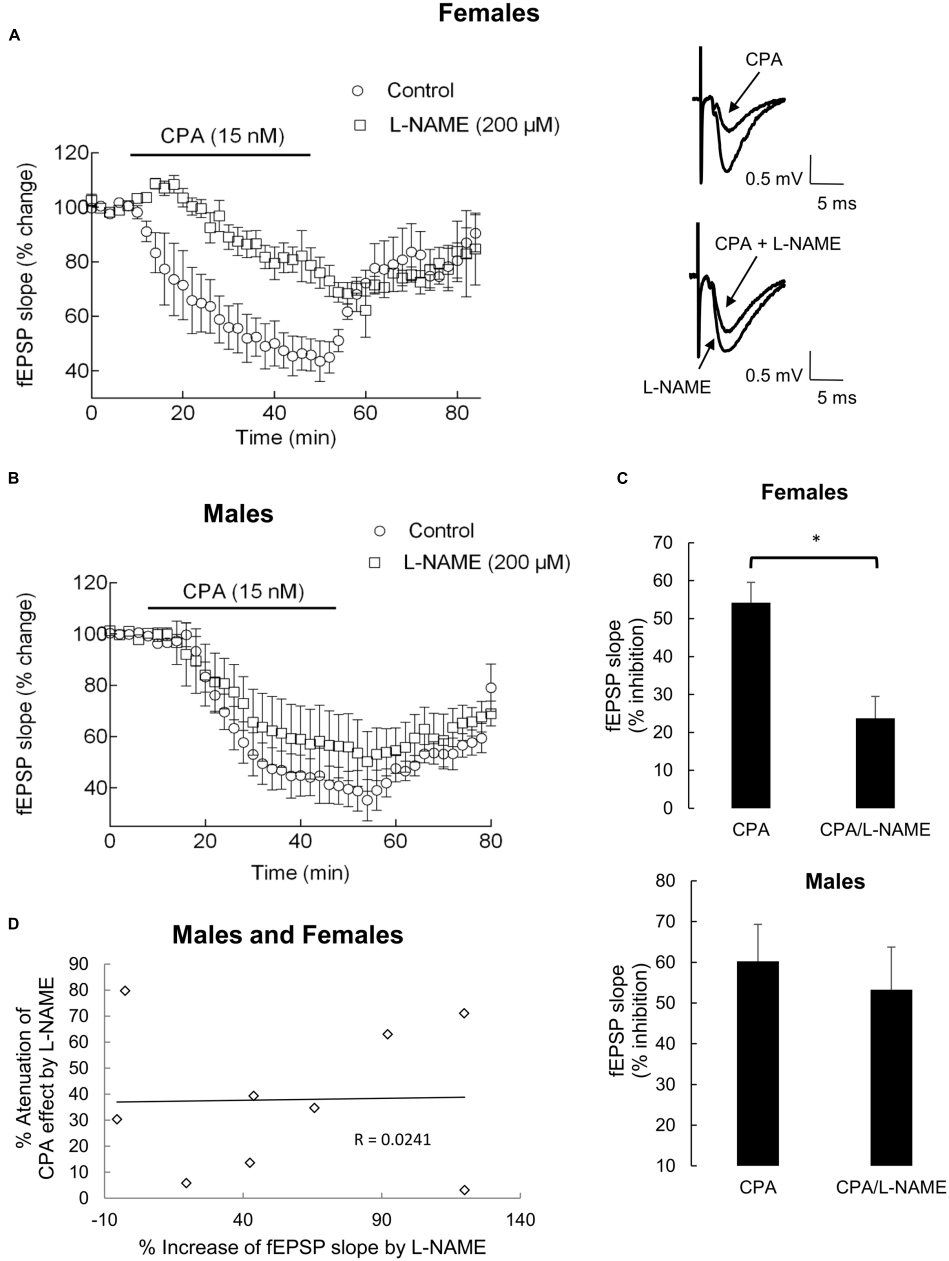
**Nitric oxide (NO) synthase antagonist L-NAME (200 μM) dampened CPA-induced inhibition of synaptic transmission in hippocampal slice of female **(A)** and male **(B)** rats.** CPA (15 nM) was first applied for 40 min (control time course) and washed-out for a minimum of 40 min more. L-NAME (200 μM) was then applied for 30 min and throughout the second application of CPA (test time course). Control and test time courses were performed in the same hippocampal slice. **(A,B)**: Superimposition of timecourses obtained for female **(A**, left) and male **(B)** rats; each point in the ordinates corresponds to the mean ± SEM of four experiments for males rats and five experiments for female rats; in each experiment a point corresponds to the average of eight consecutive fEPSP slopes; the time-distance between points corresponds to 2 min; 100% represents average fEPSP slope recorded for 10 min before applying CPA under each testing condition. Representative traces of averaged fEPSPs recorded before and after 40 min application of CPA, in the absence (upper) or in the presence (lower) of L-NAME, are shown for females (**A**, right). As one may observe in the superimposition of time courses, the effect of CPA was dampened by the presence of L-NAME in females, without interfering with the washout of CPA. **(C)**: Comparison between average of percentage inhibition produced by CPA in the absence (left) or in the presence (right) of L-NAME is shown for females (upper graphic) and males (lower graphic); the bars represented the average ± SEM after stabilization of the inhibitory effect of CPA. ^∗^*P* < 0.008 (Paired Student’s *t*-test). **(D)**: Absence of correlation between the effect of L-NAME per se on fEPSP slope and the % attenuation of the effect of CPA by L-NAME (*P* > 0.05), for males and females together.

To evaluate whether production of NO, an activator of soluble guanylyl cyclase, contributes to the inhibition of fEPSPs caused by adenosine A_1_ receptor activation, the effect of CPA in the absence and in presence of L-NAME, a NOS inhibitor (Lessmann et al., 2011), was compared in the same slice. In these experiments, L-NAME was applied to the preparations at least 40 min after starting to washout the first CPA application and 30 min before addition of the second CPA application, which was added to the slices only after stabilization of fEPSPs slope in the presence of L-NAME. The slope of fEPSPs for the last 10 min before application of CPA was taken as 100%, to allow comparison between the effects of CPA in the two conditions. Male and female animals were used and data analyzed separately since we previously found that adenosine A_1_ receptor modulation of the NO/cGMP pathway in the hippocampus depended on gender (Serpa et al., 2014). As illustrated in **Figure 2**, in the presence of L-NAME (200 μM) the inhibitory action of CPA on fEPSPs was clearly attenuated in slices taken from female rats. CPA (15 nM) alone depressed the fEPSP slope by 54 ± 5%, whereas in the presence of L-NAME (200 μM) it decreased the fEPSP slope by only 23 ± 6% (*n* = 5; *P* < 0.01, paired Student’s *t*-test, compared with CPA alone; *P*w > 95%), which corresponds to a 57 ± 9% reduction (*n* = 5; *P* < 0.05 compared with zero) of the CPA effect. In experiments performed with male rats, L-NAME also showed a tendency to dampen the effect of CPA, since CPA (15 nM) alone depressed the fEPSP slope by 60 ± 9% whereas in the presence of L-NAME (200 μM) it decreased the fEPSP slope by 53 ± 10% (*n* = 4; *P* = 0.06, paired Student’s *t*-test, compared with CPA alone; *P*w = 52%). This corresponds to a 14 ± 6% (*n* = 4) decrease of the CPA effect produced by L-NAME, which was significantly lower (*P* < 0.01, Student’s *t*-test; *P*w > 85%) than the % decrease obtained in female rats (57 ± 9%, *n* = 5). L-NAME *per se*, added to the slices after the first washout of CPA, caused an excitatory effect on fEPSPs either in slices from male or female rats (range: -5.5 to 120%; average: 55 ± 16% increase, *n* = 9, males and females, *P* < 0.01 when compared with zero, Student’s *t*-test; *P*w > 85%), no significant differences between the effect in both genders being detected (*P* > 0.70, Student’s *t*-test; *P*w = 5%). Maximal effects of L-NAME on fEPSP slope were achieved within 20 min after its application to the slices, CPA being applied to the slices only after at least 30 min of L-NAME perfusion. No correlation was detected between the effect of L-NAME per se on fEPSP slope and the % attenuation of the effect of CPA (**Figure 2D**).

### Inhibition of Soluble Guanylyl Cyclase Decreased the Effect of CPA on fEPSP Slope: Dependence on Adenosine Deaminase

**FIGURE 3:**
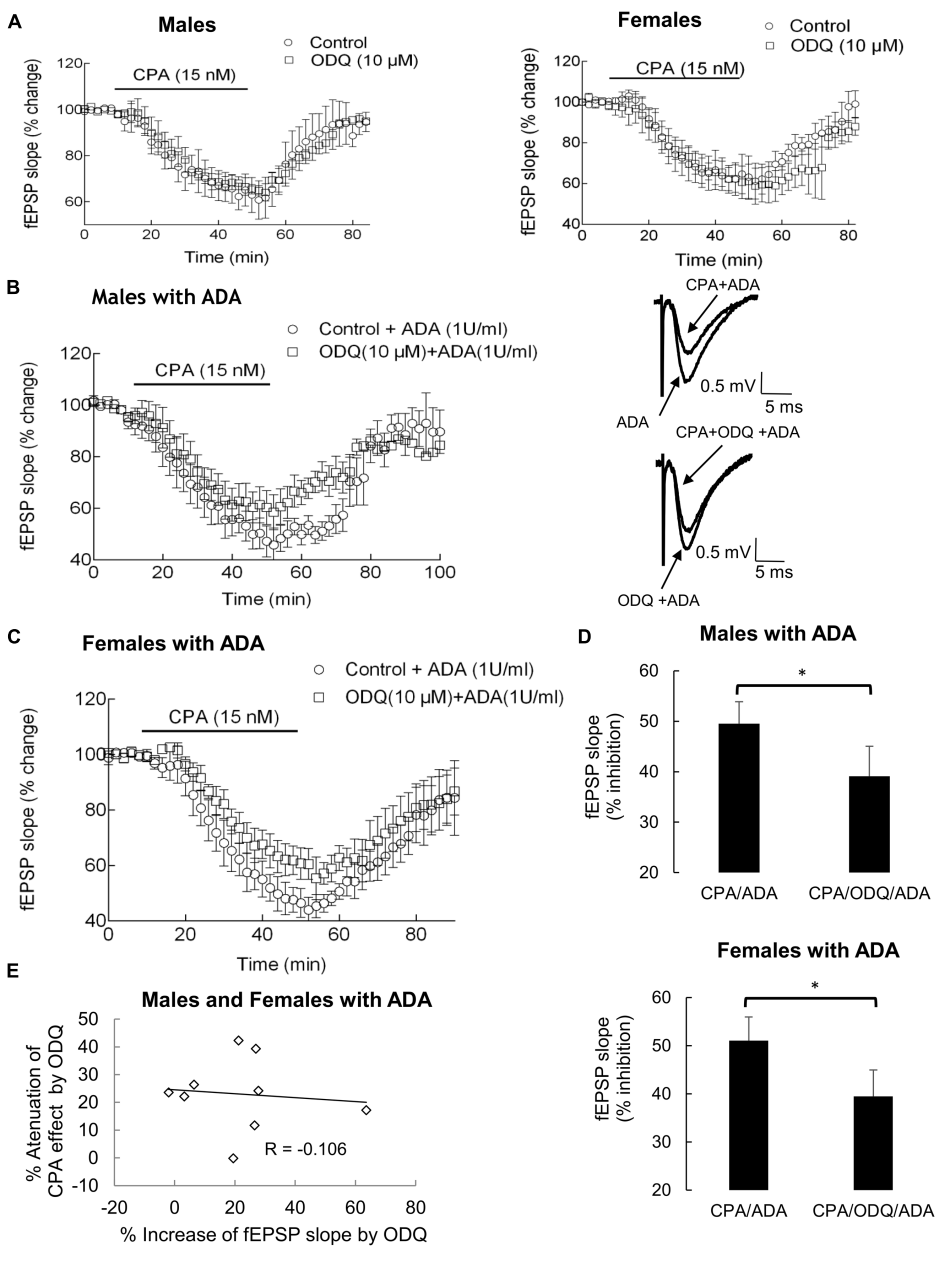
**The decrease of CPA-mediated inhibition of synaptic transmission by the sGC antagonist ODQ in males (**A**, left; **B**) and females (**A**, right; **C**) rats is unmasked by the presence of ADA **(B,C)**.** CPA (15 nM) was first applied for 40 min (control time course) and washed-out for a minimum of 40 min more. ODQ (10 μM) was then applied for 30 min and throughout the second application of CPA (test time course). Control and test time courses were performed in the same hippocampal slice. **(A–C)**: Superimposition of timecourses obtained for males **(A,B**, left) and female (**A**, right; **C**); each point in the ordinates corresponds to the average of three to five experiments performed; in each experiment a point corresponds to the average of eight consecutive fEPSP slopes; the time-distance between points corresponds to 2 min; 100% represents average fEPSP slope recorded for 10 min before applying CPA under each testing condition. Representative traces of averaged fEPSPs recorded before and after 40 min application of CPA in the presence of ADA and in the absence (upper) or in the presence (lower) of ODQ, are shown for males (**B**, right). As one may observe in the superimposition of time courses in **(A)**, the effect of CPA and washout was not modified by the presence of ODQ. The presence of ADA (1 U/ml) unmasked the dampening effect of ODQ on CPA-mediated inhibition of synaptic transmission **(B,C)** in male and female rats hippocampal slices. **(D)**: Comparison between average of percentage inhibition by CPA in the presence of ADA and in the absence (left) or in the presence (right) of ODQ is shown for males (upper graphic) and females (lower graphic); the bars represent average ± SEM after stabilization of the inhibitory effect of CPA. ^∗^*P* < 0.05 (Paired Student’s *t*-test). **(E)**: Absence of correlation between the effect of ODQ per se on fEPSP slope and the % attenuation of the effect of CPA by ODQ (*P* > 0.05), in the presence of ADA, for males and females together.

The target for NO is the soluble guanylyl cyclase, that catalyses the formation of cGMP from GTP. To evaluate if inhibition of fEPSPs caused by A_1_ receptor activation could require guanylyl cyclase activity, we next compared the effect of CPA in the absence and in the presence of ODQ, a soluble guanylyl cyclase irreversible inhibitor. As illustrated in **Figure 3A**, the presence of ODQ, at a concentration selective for guanylyl cyclase (Fragata et al., 2006), did not affect the inhibitory effect of CPA on fEPSPs recorded from hippocampal slices taken either from male or female rats. Application of ODQ alone, at least 40 min after starting the first washout of CPA and 30 min before addition of the second CPA application, increased the fEPSP slope by 17 ± 3% in males (*n* = 3; *P* < 0,05, when compared with zero, Student’s *t*-test; *P*w > 80%) and by 29 ± 4% in females (*n* = 3; *P* < 0,02, when compared with zero, Student’s *t*-test; *P*w > 90%).

We then questioned if endogenous adenosine was interfering with A_1_ receptor activity in a way that it was masking the effect of ODQ on CPA-mediated inhibition of the fEPSP slope. To assess that issue we repeated the above experiments but in the presence of ADA, that metabolizes adenosine into inosine (see Lopes et al., 1999). ADA (1 U/ml) was present throughout the experiment, thus during the first and second applications of CPA. The inhibitory effect of CPA (15 nM) on fEPSPs slope obtained in the absence of ADA, 37 ± 4% (*n* = 7, males and females), was increased to 50 ± 3% (*n* = 9, males and females) in the presence of ADA (*P* < 0.02, Student’s *t*-test; *P*w = 70%). In the experiments performed with ADA present, ODQ (10 μM) attenuated the inhibitory effect of CPA (15 nM) on the fEPSP slope, the attenuation being statistically significant either in males or females. Indeed, in the presence of ODQ, the effect of CPA was attenuated (**Figures 3B–D**) from 50 ± 4% to 39 ± 6% in males (*n* = 5; *P* < 0.02; paired Student’s *t*-test; *P*w > 80%) and from 51 ± 5% to 39% ± 6% in females (*n* = 4; *P* < 0.05; paired Student’s *t*-test; *P*w = 80%), corresponding to an attenuation of CPA effect of 23 ± 6% in males and 23 ± 7% in females. The effect of CPA (15 nM), as well as the dampening of the effect of CPA (15 nM) by ODQ (10 μM), were identical in both genders (*P* > 0.05, Two-Way ANOVA, post hoc LSD test). ODQ (10 μM) per se but in the presence of ADA (1 U/ml) increased the fEPSP slope by a similar magnitude (range: -2.0 to 64%, average: 21 ± 6%, *n* = 9, males and females, *P* < 0.01 when compared with zero, Student’s *t*-test; *P*w > 80%) as in the absence of ADA, no significant differences being detected in the magnitude of the effect in hippocampal slices taken from male or female rats (*P* > 0.3, Student’s *t*-test; *P*w = 20%). Furthermore, the effect of ODQ per se in the presence of ADA didn’t differ (*P* > 0.85, student’s *t*-test; *P*w = 5%) from its effect in the absence of ADA (23 ± 3% increase, *n* = 6, males and females). No correlation was detected between the magnitude of the increase of fEPSP slope caused by ODQ and the % attenuation of the effect of CPA caused by this guanylyl cyclase inhibitor (**Figure 3E**).

### The Inhibitory Effect of CPA on fEPSP Slope Is Attenuated by a Protein Kinase G (PKG) Inhibitor

**FIGURE 4:**
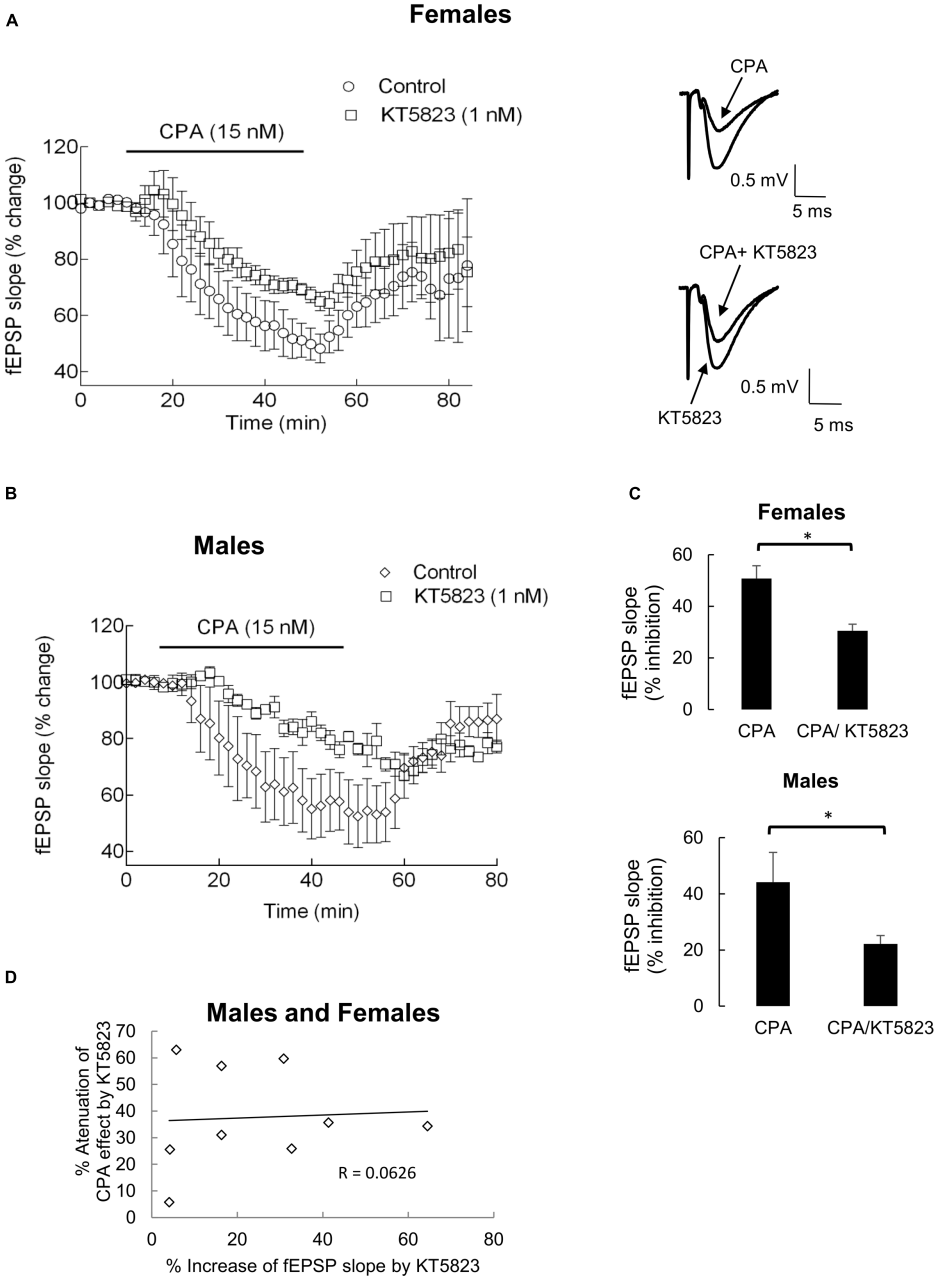
**KT5823 attenuated the inhibition of synaptic transmission induced by CPA in the CA1 area of hippocampal slices of female **(A)** and male **(B)** rats.** CPA (15 nM) was first applied for 40 min (control time course) and washed-out for a minimum of 40 min more. KT5823 (1 nM) was then applied for 30 min and throughout the second application of CPA (test time course). Control and test time courses were performed in the same hippocampal slice. **(A,B)**: Superimposition of timecourses obtained for female (**A**, left) and male **(B)** rats; each point in the ordinates corresponds to the mean ± SEM of four experiments for male and five experiments for female rats; in each experiment a point corresponds to the average of eight consecutive fEPSP slopes; the time-distance between points corresponds to 2 min; 100% represents average fEPSP slope recorded for 10 min before applying CPA under each testing condition. Representative traces of averaged fEPSPs recorded before and after 40 min application of CPA, in the absence (upper) or in the presence (lower) of KT5823, are shown for females (**A**, right). As one may observe in the superimposition of time courses, the effect of CPA decreased in the presence of KT5823, without interfering with the washout of CPA. **(C)**: Comparison between average of percentage inhibition produced by CPA in the absence (left) or in the presence (right) of KT5823 is shown for females (upper graphic) and males (lower graphic); the bars correspond to average ± SEM after stabilization of the inhibitory effect of CPA. ^∗^*P* < 0.05 (Paired Student’s *t*-test). **(D)**: Absence of correlation between the effect of KT5823 per se on fEPSP slope and the % attenuation of the effect of CPA by KT5823 (*P* > 0.05), for males and females together.

The biological actions of cGMP are predominantly due to activation of PKG. We thus evaluated whether the effect of adenosine A_1_ receptors in synaptic transmission depend on PKG activity, by comparing the effect of CPA in the absence and in the presence of a selective PKG inhibitor, KT5823. In hippocampal slices from either male (**Figures 4B,C**) or female (**Figures 4A,C**) rats, the inhibitory effect of CPA (15 nM) on fEPSPs was significantly (*P* < 0.05) attenuated in the presence of KT5823, which was used at a concentration (1 nM) known to be selective for PKG (Reyes-Harde et al., 1999). Indeed, in hippocampal slices from male rats, CPA (15 nM) alone depressed the fEPSP slope by 44 ± 11% while in the presence of KT5823 (1 nM) it decreased the fEPSP slope by 22 ± 3%, which corresponds to a 45 ± 9% attenuation of the CPA effect (*n* = 4; *P* < 0.02, compared with zero, Student’s *t*-test; *P*w > 90%). With female rats the results were similar to those obtained with male rats, since CPA (15 nM) alone depressed the fEPSP slope by 50 ± 4% whereas in the presence of KT5823 (1 nM) it decreased the fEPSP slope by 34 ± 4% (*n* = 5; *P* < 0.05, compared with CPA alone, paired Student’s *t*-test) corresponding to a 32 ± 9% decrease in CPA effect (*P* < 0.02, compared with zero, Student’s *t*-test; *P*w = 80%). Application of KT5823 (1 nM) alone for 30 min, after the first washout of CPA and before the addition of CPA in its presence, increased the fEPSPs either in slices from male or female rats (range: 4.0 to 65%; average: 24 ± 7% increase, *n* = 9, males and females, *P* < 0.01 when compared with zero, Student’s *t*-test; *P*w > 85%), no significant differences being detected in the magnitude of the effect in hippocampal slices taken from male or female rats (*P* > 0.85, Student’s *t*-test; *P*w = 5%). Furthermore, no correlation was observed between the magnitude of the effect of KT5823 per se and the % attenuation of the effect of CPA by KT5823 (**Figure 4D**).

## Discussion

The main finding in the present work is that the NOS/sGC/PKG pathway plays a role in the adenosine A_1_ receptor-mediated inhibition of excitatory synaptic transmission in the hippocampus. This is in accordance with the recent finding that adenosine A_1_ receptor activation increases cGMP formation at the hippocampus (Serpa et al., 2014) where cGMP decreases neurotransmitter release (Nordström and Bartfai, 1981).

Evidences of interaction between adenosine A_1_ receptor and the NOS/sGC/PKG pathway have been previously reported. It was shown that in the hippocampus (Saransaari and Oja, 2004) and ventral striatum (Fischer et al., 1995) the release of adenosine is markedly potentiated by NO donors and inhibited by NOS inhibitors, suggesting that endogenous NO modulates adenosine release. In fact, it has been suggested that the inhibition of field EPSPs caused by NO at the hippocampus is mediated by NO-induced release of adenosine which subsequently acts to depress neurotransmission through A_1_ receptors (Fallahi et al., 1996). Accordingly, NO-mediated inhibition of synaptic transmission in hippocampal slices was blocked by an adenosine A_1_ receptor antagonist (Boulton et al., 1994; Broome et al., 1994). However, this effect of NO might not be mediated by sGC activation since inhibition of soluble guanylyl cyclase did not affect the inhibitory effect of NO on synaptic transmission (Arrigoni and Rosenberg, 2006). Regarding synaptic plasticity, most studies indicate that it is facilitated by activation of the NOS/sGC/PKG pathway (Bohme et al., 1991; Arancio et al., 1995; Boulton et al., 1995; Reyes-Harde et al., 1999), while A_1_ receptors usually attenuate synaptic plasticity induced by electrical stimulation (de Mendonça and Ribeiro, 2000). However, activation of adenosine A_1_ receptor together with the simultaneous increase in cGMP concentration elicited by zaprinast, suffices to induce chemical LTD (Santschi et al., 2006). On the other hand, the inhibitory effect of A_1_ receptor on basal synaptic transmission is mimicked by activation of the NOS/sGC/PKG pathway, since stimulating NOS, activating soluble guanylyl cyclase or elevating concentrations of intracellular cGMP depressed synaptic transmission in CA_1_ hippocampal neurons (Lei et al., 2000). In accordance with an inhibitory action of the NOS/sGC/PKG pathway on basal synaptic transmission are the present observations that inhibitors of NOS (L-NAME), sGC (ODQ), and PKG (KT5823) facilitate fEPSPs.

Adenosine A_1_ receptor-mediated inhibition of synaptic transmission can be potentiated by an NO donor, an action blocked by the sGC antagonist ODQ, suggesting a facilitatory effect of cGMP on the adenosine A_1_ receptor effect at the hippocampus (Fragata et al., 2006). Concerning the role of cGMP on adenosine A_1_ receptor-dependent activity, first evidence comes from a recent study showing that the inhibitory effect of peripheral adenosine A_1_ receptors on inflammatory hypernociception was blocked by sGC and PKG inhibitors (Lima et al., 2010). We now report that when the activity of components of the NOS/sGC/PKG pathway was blocked by the corresponding selective inhibitors (L-NAME, ODQ, and KT5823) the inhibitory effect of adenosine A_1_ receptors on hippocampal synaptic transmission was attenuated, thus indicating that the cGMP signaling cascade contributes in part for the inhibitory action of adenosine at excitatory synapses. The fact that removal of endogenous adenosine with ADA did not affect the potentiating effect of ODQ per se on fEPSP and, on another hand, the absence of correlation between the magnitude of the increase of fEPSP slope caused by either ODQ, L-NAME, or KT5823 and the % attenuation of the effect of CPA caused by these inhibitors also strongly suggest that the potentiating effects of ODQ, L-NAME, or KT5823 *per se* on fEPSP slope are not mediated by dampening of tonic activation of A_1_ receptors by endogenous adenosine. Interestingly, we found that the dampening of the A_1_ receptors on synaptic transmission by the NOS inhibitor, L-NAME, was stronger in females than in male rats, suggesting that the NOS-mediated inhibition of synaptic transmission elicited by adenosine A_1_ receptor plays a more relevant role in females. This result is in agreement with our recent finding that adenosine A_1_ receptor-mediated activation of the NOS/sGC pathway depends on gender, the A_1_ effect being stronger in females than in males rats (Serpa et al., 2014). Although the dampening by L-NAME of the A_1_ receptor-mediated inhibition of fEPSP was stronger in females, the stimulatory effect of L-NAME alone on fEPSP did not differ across gender, suggesting that while basal NOS activity inhibits synaptic transmission equally in males and females rats, the role of NOS in mediating A_1_ receptor inhibition of fEPSP is more relevant in females.

Curiously, the attenuation of the CPA effect on synaptic transmission by the sGC inhibitor, ODQ, was only evident in the presence of ADA. The absence of attenuation by ODQ of the effect of CPA in the absence of ADA, could be consequence of A_1_ receptor occupation with endogenous adenosine which might be enough to produce maximal activation of sGC, preventing further activation of sGC with the exogenous agonist. Evidence that endogenous adenosine was interfering with the CPA effect came from the observation that the presence of ADA increased the CPA inhibitory effect on fEPSPs. Therefore, the removal of endogenous adenosine by ADA would unmask the sGC-mediated effect of CPA on fEPSPs. Indeed, to detect the facilitatory action of adenosine A_1_ receptor agonists on cGMP formation it is also necessary to add ADA to the incubation medium (Serpa et al., 2014). However, some actions of ADA on the A_1_ receptor may be independent of endogenous adenosine removal. In fact, previous studies showed that extracellular ADA binds to adenosine A_1_ receptors, increasing its affinity toward agonists, thus acting as a co-stimulatory molecule to facilitate specific signaling events, independently from its enzymatic activity (reviewed in Franco et al., 2005). Interestingly the dampening of the CPA effect on synaptic transmission by NOS blockade did not required the presence of ADA. On the other hand, the dampening of the CPA effect by NOS blockade was stronger in females than in males, while the dampening of the CPA effect by blockade of sGC, in the presence of ADA, didn’t depend on gender. These results suggest that the NOS-mediated inhibition of synaptic transmission by adenosine A_1_ receptor might not involve exclusively activation of sGC by NO. In fact both sGC-mediated and sGC-independent inhibition of glutamate release by NO from hippocampal nerve terminals has been described (Sequeira et al., 1999).

It is known for a long time that A_1_ receptors inhibit synaptic transmission at the hippocampus by activating G_i/o_ proteins. This effect of adenosine A_1_ receptor is primarily consequence of presynaptic inhibition of neurotransmitter release. One of the mechanisms by which adenosine A_1_ receptor inhibits neurotransmitter release involves reduction of Ca^2+^ entry trough N-type calcium channels (Wu and Saggau, 1994), while a decrease of cAMP levels does not seems to mediate the A_1_ receptor-dependent inhibition of neurotransmitter release (see Fredholm and Dunwiddie, 1988). However, blockade of N-type calcium channels only partially attenuates the inhibitory effect of A_1_ receptor on synaptic transmission, suggesting that other mechanisms might be involved (Fredholm and Dunwiddie, 1988). On the other hand, activation of p38 MAPK by adenosine A_1_ receptors has also been shown to be involved in the inhibitory activity of A_1_ receptors on synaptic transmission (Brust et al., 2006). Interestingly, both activation of p38 MAPK (Browning et al., 2000) and N-type calcium channels (D’Ascenzo et al., 2002) by cGMP-dependent mechanisms has been described. If inhibition of neurotransmission by adenosine A_1_ receptors-mediated stimulation of NOS/sGC/PKG pathway involves N-type channels inhibition and/or p38 MAPK activation or constitutes another parallel mechanism mediating the presynaptic inhibitory effect of adenosine A_1_ receptors on synaptic transmission in the hippocampus, is an interesting issue and deserves future investigation.

## Conclusion

The present work shows that blocking the NOS/sGC/PKG signaling pathway dampens adenosine A_1_ receptor-mediated inhibition of synaptic transmission, revealing that control of synaptic activity by adenosine A_1_ receptors partially depends on activation of the cGMP signaling cascade, which may operate in a concerted way with other A_1_ receptor coupled pathways to fine-tune neuronal excitability.

## Author Contributions

IP contributed to the design of the work and to the acquisition, analysis, and interpretation of data for the work; the author also made the first draft of the manuscript. AS contributed to the conception and design of the work and to the acquisition, analysis, and interpretation of data for the work; the author also contributed to the first draft of the manuscript. AMS contributed to the conception and design of the work and to the interpretation of data for the work; the author also revised the manuscript. JC contributed to the conception and design of the work and to the analysis and interpretation of data for the work; the author also revised the manuscript. All authors gave their final approval of the version of the manuscript to be published and agree to be accountable for all aspects of the work in ensuring that questions related to the accuracy or integrity of any part of the work are appropriately investigated and resolved.

## Conflict of Interest Statement

The authors declare that the research was conducted in the absence of any commercial or financial relationships that could be construed as a potential conflict of interest.
